# Tryptophan Seed Treatment Improves Morphological, Biochemical, and Photosynthetic Attributes of the Sunflower under Cadmium Stress

**DOI:** 10.3390/plants13020237

**Published:** 2024-01-15

**Authors:** Mujahid Hussain, Rehana Kaousar, Sharafat Ali, Changfeng Shan, Guobin Wang, Shizhou Wang, Yubin Lan

**Affiliations:** 1 College of Agricultural Engineering and Food Science, Shandong University of Technology, Zibo 255022, China; mujahidagr@gmail.com (M.H.);; 2 Department of Botany, College of Life Sciences, Government College University, Allama Iqbal Road, Faisalabad 38000, Pakistan; 3 National Center for International Collaboration Research on Precision Agricultural Aviation Pesticides Spraying Technology (NPAAC), Ministry of Science and Technology, College of Electronics Engineering, South China Agricultural University, Guangzhou 510642, China

**Keywords:** heavy metal stress, metabolites, photosynthetic pigments, seed treatment, tryptophan, sunflower

## Abstract

Tryptophan, as a signal molecule, mediates many biotic and environmental stress-induced physiological responses in plants. Therefore, an experiment was conducted to evaluate the effect of tryptophan seed treatment in response to cadmium stress (0, 0.15, and 0.25 mM) in sunflower plants. Different growth and biochemical parameters were determined to compare the efficiency of the treatment agent. The results showed that cadmium stress reduced the growth attributes, including root and shoot length, dry and fresh weight, rate of seed germination, and the number of leaves. Cadmium stress also significantly reduced the contents of chlorophyll *a*, *b*, and total chlorophyll, carotenoid contents, phenolics, flavonoids, anthocyanin, and ascorbic acid. Whereas cadmium stress (0.15 and 0.25 mM) enhanced the concentrations of malondialdehyde (45.24% and 53.06%), hydrogen peroxide (−11.07% and 5.86%), and soluble sugars (28.05% and 50.34%) compared to the control. Tryptophan treatment decreased the effect of Cd stress by minimizing lipid peroxidation. Seed treatment with tryptophan under cadmium stress improved the root (19.40%) and shoot length (38.14%), root (41.90%) and shoot fresh weight (13.58%), seed germination ability (13.79%), average leaf area (24.07%), chlorophyll *b* (51.35%), total chlorophyll (20.04%), carotenoids (43.37%), total phenolic (1.47%), flavonoids (19.02%), anthocyanin (26.57%), ascorbic acid (4%), and total soluble proteins (12.32%) compared with control conditions. Overall, the tryptophan seed treatment showed positive effects on sunflower plants’ growth and stress tolerance, highlighting its potential as a sustainable approach to improve crop performance.

## 1. Introduction

The sunflower (*Helianthus annuus* L.) belongs to the family Asteraceae, the fourth-largest oil-seed crop in the world, which is used as fuel. Seeds are consumed as food, salad dressings, and margarine for cooking and oil extraction [1]. The production of sunflowers in the world in 2022–2023 was carried out on an area of 28.01 million hectares with an average yield production of 1.87 metric tons ha^−1^ [2]. Studies have shown that abiotic stress, including heavy metals, is the main obstacle that negatively affects sunflower growth, seed germination, photosynthesis attributes, and nutritional values; however, the crop response to this stress may vary with the duration of metal exposure, metal type, and soil type [3]. Heavy metals such as Cadmium (Cd) are toxic elements that threaten soil, water, and the environment and result from rapid urbanization and industrialization worldwide [4]. These are added to the environment through human and anthropogenic activities and are hazardous to plants even at low levels [5,6]. Even the low levels of Cd mobility in plants cause adverse effects on plant growth and metabolism [7]. Numerous studies have shown that this metal may prevent root development, restrict root growth, decrease seed germination, reduce the number of plant leaves, and cause plant mortality [8]. Plant physiological issues, particularly those brought on by elevated oxidative stress and excessive formation of reactive oxygen species (ROS), may be exacerbated by cadmium [9]. It was found that plants in contaminated soils use a variety of coping mechanisms and Cd stress management techniques [10]. The techniques can be roughly divided into two types: avoidance, restricting metal intake, and tolerance, which involves storing and accumulating potentially dangerous metals in a specific part of the plant depending on the various species [11]. The introduction of Cd into plant cells activates the plant’s defense mechanism. Numerous signaling mechanisms activate the defense system, including the calcium calmodulin pathway, excessive ROS generation, and phytohormone synthesis [12].

Seed treatment is a technique broadly used by seed scientists to improve seed vigor, tolerance, and germination potential. Different treatment techniques activating the pre-germination metabolism can be applied depending on the seed physiology, morphology, and plant species. This physiological process occurs during seed inhibition, including the seed repair response (activation of antioxidant mechanisms and DNA repair pathways), which is essential to preserve genome integrity, ensuring proper germination and seedling development [13]. Recent developments in biological and agricultural science disciplines have been made, such as molecular breeding integrative multi-omics and advanced agronomical techniques involved in heavy metal tolerance. However, these are less popular among the farming community and are limited to the lab conditions. Therefore, seed treatment is a versatile and promising heavy metal stress tolerance approach. Seed treatment with plant growth regulators (tryptophan, salicylic acid, gibberellins, and auxins) and organic and inorganic salts (proline, CaCl_2_, and Mg (NO_3_)_2_) showed promising results, countering the Cd effect [14]. This activation causes the production of numerous molecules and substances, such as calcium and nitric oxide [10]. Glutathione is a precursor for phytochelatins [15] and proline [16]. These procedures might lead to Cd chelation, decreased oxidative stress, and lessened tissue damage in plants. Modifications to molecules affect cadmium mitigation, tolerance, and adaptability. Several genes were discovered to be overexpressed in response to Cd exposure. Gene overexpression controls Cd absorption, transit from root to shoot, accumulation, sequestration, and detoxification [17]. We can also observe seed treatment technology for Cd stress tolerance at different plant growth stages such as molecular, biochemical, and physiological levels [18]. It is therefore proposed to investigate how tryptophan affects the possible over-accumulation of metabolic products from the phenylpropanoid pathway in sunflowers (*Helianthus annuus* L.), as well as the possibility that tryptophan can help the crop to cope with stress.

Tryptophan is a widely recognized amino acid essential for the growth of plants under typical ambient circumstances and various abiotic stresses. L-tryptophan is a physiological precursor to melatonin in higher plants [19]. It has a crucial role as an osmolyte compound that can control the transfer of different ions as nutrients, regulate stomatal opening, and help mitigate the negative impact of heavy metals [20]. Additionally, tryptophan may improve several biochemical procedures by influencing plant development, differentiation, and the ability to absorb nutrients and water [21]. It was demonstrated that L-tryptophan significantly affects plants’ development and productivity compared to other growth hormones [22]. Tryptophan is an excellent peptide. It could function as an ion transport regulator known as an osmolyte, which regulates stomata opening and eliminates heavy metal toxicity [20]. Auxin levels increase in plant tissues after an exogenous treatment [23]. There are several ways to provide tryptophan to plants, such as soil application [24], foliar spray [25], and seed treatment [26]. Exogenous tryptophan has active auxin synthesis [27].

Several techniques have been used to deliver tryptophan to plants, including seed treatment, foliar spraying and soil treatment. Tryptophan supplied exogenously is actively converted to auxin [28,29]. When applied to plants, the quantity of auxin produced from tryptophan changes intra- and interspecifically [30]. Jiang et al. [31] investigated the effects of foliar tryptophan on *Brassica oleracea* under Cd stress and concluded that Cd stress declined shoot and root fresh weight, which was then increased by 25 and 120% by applying tryptophan. In another research, the results revealed that *Eucalyptus gomphocephala* plants exposed to high concentrations of Cd had the lowest vegetative growth and photosynthetic parameters. In contrast, plants treated with L-tryptophan showed an increase in photosynthetic pigments, carbohydrate, and proline contents in controlled or Cd stress conditions [32]. The combined application of L-tryptophan and the plant growth-promoting rhizobacteria improved the translocation factor and the bioconcentration factor in sunflower shoots and increased lead (PbCl_2_) removal efficiency by 22% [33]. Numerous studies have found that tryptophan spraying improves crop growth and production [34]. It was shown that combining tryptophan with auxin-producing microorganisms enhances the stimulatory impact of tryptophan [35]. Due to the increasing Cd levels in farmland soils, many crops face Cd pollution. However, studies on the role of a tryptophan seed treatment on sunflower crops under Cd stress are limited. It is of great importance to reduce Cd toxicity in crops. It was summarized that tryptophan has a particular function in plant responses to Cd stress in various crops. Therefore, in this study, we evaluated the effect of tryptophan as an amino acid seed treatment on germination traits under Cd stress conditions compared with a non-tryptophan treatment in sunflowers. We further performed a biochemical analysis and exposed the possible mechanism of the beneficial effects of tryptophan. Our study provides information regarding the better utilization of tryptophan to increase crop growth by resisting Cd stress.

## 2. Materials and Methods

### 2.1. Plant Material and Experimental Layout

The pot research was conducted at the Linzi Research Center of Shandong University of Technology, Shandong, China. A hybrid sunflower cultivar SH363 obtained from Sanrui Agritec Co., Ltd. Beijing, China. was used in this experiment. The experiment was arranged in a Complete Randomized Design (CRD) followed by three replications. The experimental treatments consisted of 2 levels of tryptophan seed treatment—non-treated (control), seed treatment with tryptophan solution (1%) 12 h before sowing, and 3 levels of Cd (CdCl_2_) concentrations (0, 0.15, and 0.25 mM kg^−1^). The seed pre-germination test was performed using the sinking method before treating it with tryptophan. Ten tested viable seeds were grown in plastic pots filled with 10 kg of soil each. The soil used in the experiment was taken from the experimental site with a depth of 0–30 cm. Soil texture was clay loam, pH was 8.0, organic content was 15.6 g kg^−1^, 1.15 g kg^−1^ of total nitrogen, 42.8 mg kg^−1^ of available P, and 138 mg kg^−1^ of available K. The experimental soil was air-dried and homogenized before use and then contaminated with Cd through uniform spraying according to the treatments. Tryptophan-treated seeds were sown in pots under natural light at 28/20 °C (day/night) in a temperature-controlled glasshouse and were irrigated up to field capacity until the experiment was terminated. After taking the germination data, the number of plants was reduced to 5 by thinning. Plants were harvested 52 days after sowing at the flowering stage, and data for various morpho-physiological and biochemical properties were documented.

### 2.2. Morphological Attributes

The plant’s shoot and root length were measured using a centimeter tape or a scale. Plant fresh shoot and root weight was measured in grams and recorded using a digital weighing scale for later examination. After washing them with tap water, cutting was employed to properly remove the shoots from the roots. Shoots and roots of the plant were kept in the open air for drying for one week; after that, the dry weight was recorded. Seed germination was tested after 12 days of sowing. The germinated seeds were counted from each pot, and the germination percentage was calculated by the total of 10 viable sown seeds. The length and width of the whole plant’s leaves were measured after cutting the plant using a centimeter tape or a scale. After calculating the whole plant leaf area, an average was taken to obtain a single leaf area/cm^2^ for comparison with various treatments. Each leaf area was calculated using the following formula:

Leaf area (cm^2^) = (leaf length) × (leaf width) × 0.75 (correction factor)

### 2.3. Photosynthetic Pigment Determination

The amount of chlorophyll *a*, *b*, and carotenoid contents of 45-day-old leaves was measured using the Arnon [36] approach. Fresh leaf material of 0.5 g was crushed down into minute fragments in the form of chopped material and then added to 10 mL of acetone (80%). At 4 °C, this mixture was preserved. Centrifugation was performed in the centrifugation apparatus at 10,000 rpm for 5–10 min. Following supernatant separation, spectrophotometer readings at 480, 663, and 645 nm were taken for Chl *a*, *b,* and carotenoids. Chlorophyll and carotenoid contents were calculated using the formula described in [37].

Chlorophyll *a* (mg/g) = [12.7 (OD 663) − 2.69 (OD 645)] × V/1000 × W

Chlorophyll *b* (mg/g) = [22.9 (OD 645) − 4.68 (OD 663)] × V/1000 × W

Total chlorophyll (mg/g) = [20.2 (OD 645) + 8.02 (OD 663)] × V/1000 × W

Carotenoids = [A Car/Em × 100], Where Em × 100 = 2500

A Car = [(OD 480) + 0.114 (OD 663) − 0.638 (OD 645)]/2500

V is the extract volume (mL), W is the weight of the fresh leaf tissue (g), and OD is optical density.

### 2.4. Analysis of Biochemical Parameters

#### 2.4.1. Malondialdehyde (MDA) Content Estimation

Heath and Packer [38] described the method for estimating MDA content. A total of 0.1 g of fresh plant leaf material was purposefully crushed to homogenize with 1 mL of TCA (5%). The homogenized mixture was then centrifuged at 12,000 rpm for 10 min. Then, 1 mL of plant extract was combined with 1 mL of TCA (20%) containing 0.5% thiobarbituric acid (TBA). The resultant mixture was then maintained for 30 min in a water bath at 95 degrees before being centrifuged for 5 min at 7500 rpm. The absorbance of produced sample was measured at 532 nm and 600 nm. TCA (5%) absorbance values were measured at both wavelengths as a blank. By deducting the absorbance value at 532 nm from the result at 600 nm, the turbidity value of the prepared sample was adjusted.

#### 2.4.2. Estimation of Hydrogen Peroxide Content

The hydrogen peroxide levels in plants were determined using the methods developed by Velikova et al. [39], i.e., total of 0.5 g of recently harvested plant leaf material mixed in a trichloroacetic acid solution at a concentration of 7 percent (*w*/*v*). Then, centrifugation for 10 to 15 min at 12,000 rpm. The supernatant was then divided. In total, 0.5 mL of phosphate buffer (7.0 pH), 0.5 mL of extract, and 1 mL of a potassium iodide solution were combined. An ultraviolet-visible spectrophotometer measured the reaction mixture’s absorbance after a vortex at 390 nm.

### 2.5. Laboratory Analysis of Osmoprotectant

#### 2.5.1. Phenolic Contents Determination (mg/g FW)

Julkunen-Tiitto [40] developed the method to determine the total phenolic content. Leaf material of 0.5 g in weight was dissolved in an acetate solution (80%). Leave the mixture alone for one night as the leaf contents are integrated into the solution. The Folin Ciocalteu phenolic reagent, 2.0 mL of distilled water, 2.0 mL of Na_2_CO_3_ 20% solution, and 0.1 mL of acetone leaf extract were combined to generate a reaction mixture. Distilled water (d H_2_O) 5 mL was added. Vortex the mixture once again for a further 15 min. Values were then captured at a wavelength of 750 nm.

#### 2.5.2. Total Flavonoids (mg/g FW)

The aluminum chloride colorimetric method developed by Zhishen et al. [41] was used to determine the flavonoids. In which, 1 mL of the supernatant solution was combined with 4 mL of distilled water and 0.6 mL of NaNO_2_ (5%). After five minutes, 10% AlCl_3_ was added in a volume of 0.5 mL. Then, 2 mL of NaOH (1 M) was added, and H_2_O was used to increase the volume to 10 mL. The absorbance was measured with a spectrophotometer at 510 nm.

#### 2.5.3. Anthocyanin Determination (mg/g FW)

The anthocyanin contents were determined by following the Hodges and Nozzolillo [42] method. In total, 0.1 g of leaf material was ground in 2 mL of methanol and heated for 1 h. After centrifugation, the wavelength was checked at 600 nm using a spectrophotometer.

#### 2.5.4. Ascorbic Acid Determination (mg/g FW)

The ascorbic acid concentration was determined using the method published by Mukherjee and Choudhuri [43]. One gram of the plant material was mixed well with 10% trichloroacetic acid and centrifuged for 20 min at 4000 rpm. The aliquot (3.5 mL) was then mixed with 0.5 mL of a 9N sulfuric acid solution containing 1 g of 2,4-dinitrophenyl hydrazine, 0.04 g of copper sulfate, and 2 g of thiourea. The mixture was then heated for three hours at 37 °C. After cooling the solution on ice, 65% sulfuric acid was added. The mixture was maintained at 30 °C for half an hour. The mixture was then centrifuged at 15,000 rpm for 20 min. The resulting color was studied at a wavelength of 520 nm in a spectrometer.

#### 2.5.5. Total Soluble Proteins Determination (mg/g FW)

Leaf material was pulverized for protein analysis using the Bradford [44] phosphate buffer (pH 7.8) method. The leaf material was ground before being centrifuged for 15 min at the lowest temperature and 12,000 rpm (4 °C). The Bradford reagent was created using the subsequent chemical reaction and used for the analysis.

##### Preparation of Bradford Reagent

Commissive brilliant blue 0.1 g was combined with 850 mL of pure water, 100 mL orthophosphoric acid, and 50 mL of either 100% methanol or 95% ethanol. The Bradford reagent and 0.1 mL of enzyme extract were combined and then incubated for 20 min. A spectrophotometer was used to determine the reaction mixture’s absorbance at 596 nm.

#### 2.5.6. Determination of Total Soluble Sugar (TSS) (mg/g FW)

The Riazi et al. [45] technique was used to determine total soluble sugar content. For this, 0.1 mL of extract was assorted with 2 mL of anthrone substance, and then the subsequent solution was left for thirty minutes. Readings were recorded at 620 nm.

### 2.6. Statistical Analysis

The software SPSS 16.0 (SPSS, Chicago, IL, USA) was used for statistical analysis. Factorial ANOVA was used to determine the mean comparison and significant differences between all the treatments at *p* < 0.05, followed by LSD. A graphical representation of the obtained results was achieved using Origin 2021 software 9.8 (Origin Lab Co., Northampton, MA, USA).

## 3. Results

### 3.1. Root and Shoot Characteristics

**Figure 1:**
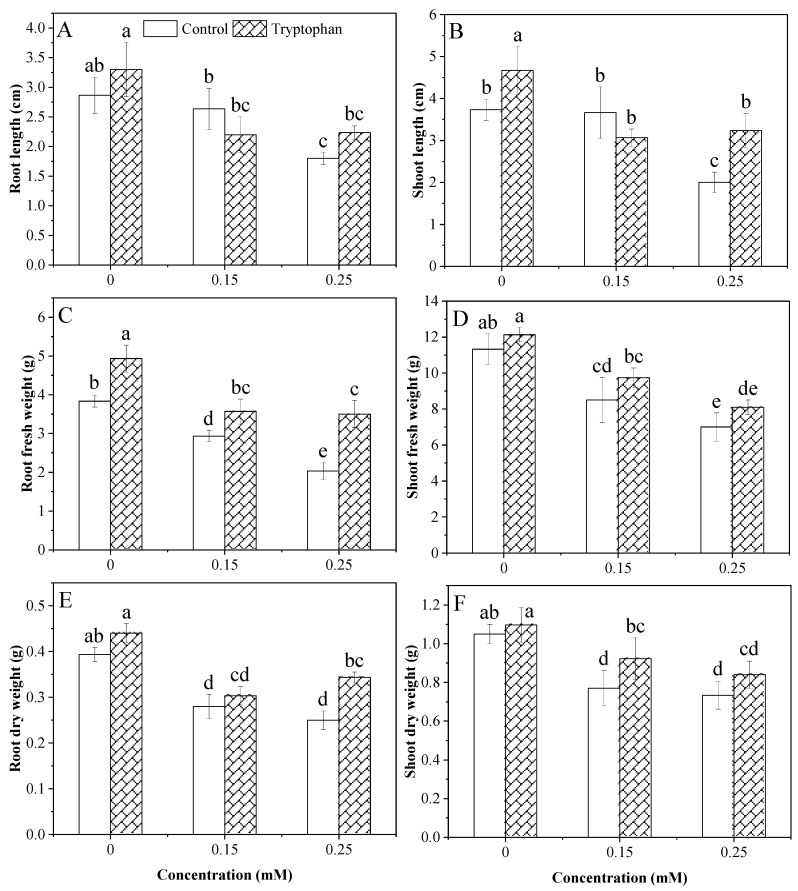
Comparative change in sunflower root and shoot morphology under cadmium levels (0, 0.15, 0.25 mM) with tryptophan (1%) seed treatment (**A**–**F**). Error bars indicate standard deviation, and the different letters indicate significant differences at *p* < 0.05 using the LSD test.

Data from the analysis of variance showed the highly significant effect of Cd stress levels. In contrast, the effect of the tryptophan seed treatment on root length and the interaction between the two factors was non-significant. The root length of both treated and untreated plants was similar under 0 mM Cd stress conditions. Cadmium stress significantly decreased root length, which was more pronounced at higher stress levels (0.25 mM). Moreover, the effect of the tryptophan seed treatment was almost negligible under both Cd levels (Figure 1A). The shoot length of both conditions (control and tryptophan seed treatment) was decreased under Cd stress. Cd stress of 0.25 mM significantly decreased the shoot length, while there was an insignificant difference between the control and the 0.15 mM levels. The tryptophan effect was found to be positive in alleviating stress in both Cd stress levels. Moreover, the interaction between these factors was significant (Figure 1B). In untreated plants, there was a decline in fresh and dry root weight as the stress was increased; hence the maximum decrease was noted at 0.25 mM of Cd stress. The tryptophan seed treatment successfully improved fresh and dry root weight under Cd stress levels. Cd stress levels and the tryptophan seed treatment had a highly significant effect on fresh and dry root weight, while the interaction between these two factors was observed as insignificant (Figure 1C,E). A highly significant effect on shoot fresh and dry weight was observed for the level of Cd stress and a substantial effect was observed with the tryptophan seed treatment. Moreover, a non-significant interaction between these two factors was observed (Table 1). Fresh and dry biomass was decreased by Cd stress (0.15 and 0.25 mM). Regarding the effect of tryptophan, it was found to be positive in alleviating stress at the lower level of stress exclusively (0.15 mM). However, at higher stress levels (0.25 mM), the tryptophan seed treatment failed to have any effect (Figure 1D,F).

### 3.2. Photosynthetic Pigments

**Figure 2:**
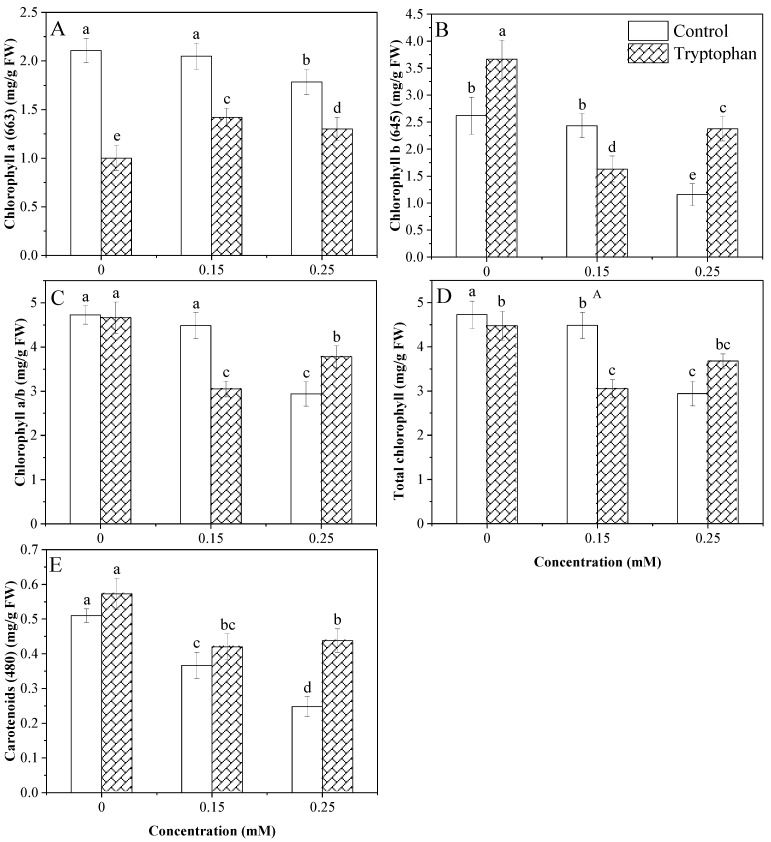
Comparative change in photosynthetic pigments under cadmium levels (0, 0.15, 0.25 mM) with tryptophan (1%) tryptophan seed treatment (**A**–**E**). Error bars indicate standard deviation, and the different letters indicate significant differences at *p* < 0.05 using the LSD test.

Data analysis showed a non-significant effect of Cd levels on chlorophyll *a* and the significant effect of the tryptophan seed treatment. Moreover, the interaction between these factors was significant. Cadmium stress (0.25 mM) significantly decreased leaf chlorophyll *a*. As regards the effect of tryptophan, it was found to be almost negligible (Figure 2A). Chlorophyll *b* was significantly reduced by both Cd stress levels. However, the effect of the 0.15 mM stress level was insignificant compared to the 0.25 mM stress level. The effect of the tryptophan seed treatment was found to be positive in alleviating stress in two stress levels (0 and 0.25 mM). The analysis of variance showed a significant difference between the effects of the Cd stress levels, the tryptophan seed treatment, and the interaction of these factors (Figure 2B). For the carotenoid contents, total chlorophyll, and the ratio of chlorophyll *a*/*b*, a highly significant effect relating to the Cd stress level was observed. In contrast, the effect of the tryptophan seed treatment on total chlorophyll and carotenoid content was significant, but a non-significant effect was observed on the ratio of chlorophyll *a*/*b*. Moreover, a significant interaction was observed between the two factors for these parameters (Table 1). Cadmium stress (0.25 mM) decreased chlorophyll *a*/*b* ratio, total chlorophyll, and carotenoid contents, which was alleviated by tryptophan seed treatment to some extent (Figure 2C–E).

### 3.3. Germination and Leaf Parameters

**Figure 3:**
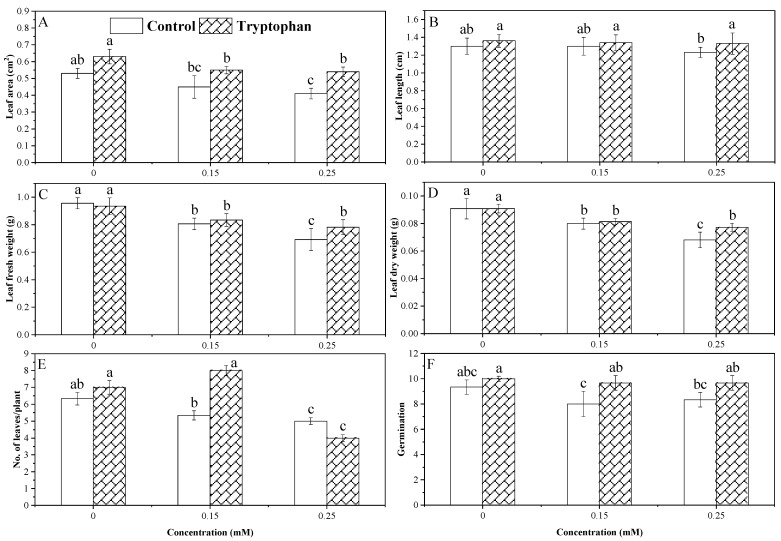
Comparative change in germination and leaf various parameters under cadmium stress levels (0, 0.15, 0.25 mM) with tryptophan (1%) seed treatment (**A**–**F**). Error bars indicate standard deviation, and the different letters indicate significant differences at *p* < 0.05 using the LSD test.

Data analysis for seed germination demonstrated a significant decrease for both Cd stress levels compared to non-stress conditions. The tryptophan seed treatment significantly increased the germination of sunflower seeds under the 0.15 mM stress level, while no significant difference was observed between the treated and the non-treated seed germination under the Cd level of 0.25 mM and the no stress condition (Figure 3F). The interaction between the two factors for this attribute was not significantly different (Table 2). For per plant leaves, a highly significant effect in relation to stress level and a substantial effect with the tryptophan seed treatment was observed. Moreover, the interaction between these factors was also significant. Cadmium stress significantly decreased the number of leaves on the sunflower plants at higher Cd stress levels. Seed treatment with tryptophan (1%) at the Cd stress level of 0.15 mM significantly increased leaf number, while the seed treatment had no influence at a Cd stress level of 0.25 mM (Figure 3E). Mean comparison values showed an increased average leaf area under cadmium stress in tryptophan seed-treated plants. Compared with non-stress conditions, seed-treated plants’ average leaf area decreased under both Cd stress levels. The results showed that tryptophan (1%) alleviated the effect of the cadmium stress in terms of average leaf area. At the higher stress level, the tryptophan seed treatment significantly increased the average leaf area of the Cd-stressed plants. Moreover, the interaction between the two factors was insignificant (Figure 3A). Data analysis for average leaf length and dry and fresh weight demonstrated a significant difference between the levels of Cd stress and the tryptophan seed treatments. Average leaf length, both fresh and dry weight, was significantly increased by the tryptophan seed treatment under 0.25 mM of Cd stress, while no effect was observed in the 0.15 mM stress level and the non-stress plants with the seed treatment. Although average leaf fresh and dry weights were reduced under both Cd stress levels compared with non-stress conditions, the reduction was statistically not different. The interaction between the two factors for these attributes was not significant (Figure 3B–D).

### 3.4. Biochemical Attributes

**Figure 4:**
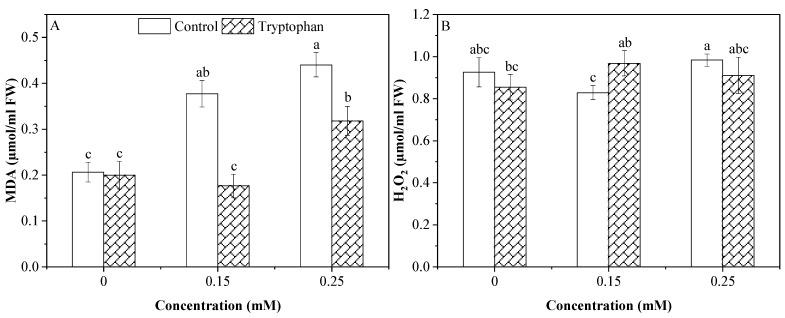
Comparative change in malondialdehyde (MDA) and hydrogen peroxide (H_2_O_2_) under cadmium stress levels (0, 0.15, 0.25 mM) with tryptophan (1%) seed treatment (**A**,**B**). Error bars indicate standard deviation, and the different letters indicate significant differences at *p* < 0.05 using the LSD test.

Statistical data of variance analysis showed a highly significant effect of Cd stress level and tryptophan seed treatment on malondialdehyde. Moreover, there was a significant interaction between the two factors. Cadmium stress significantly increased this attribute in the leaves of the sunflower plants. Tryptophan (1%) was found to be effective in lowering its value in primed plants compared to the unipositive to alleviate the stress effect in two stress levels (0.15 and 0.25 mM) (Figure 4A). For hydrogen peroxide, no significant difference was observed in tryptophan seed-treated and no-treated plants under no Cd stress and the 0.25 mM Cd level. Under lower Cd stress levels (0.15 mM), the H_2_O_2_ concentration was significantly increased in the tryptophan seed-treated plants compared to non-treated plants. Still, this was the same as the plants under no Cd stress (Figure 4B). Statistical analysis showed a non-significant difference between all Cd stress levels and the tryptophan seed treatment. At the same time, the interaction between the two factors was significantly different (Table 3).

**Figure 5:**
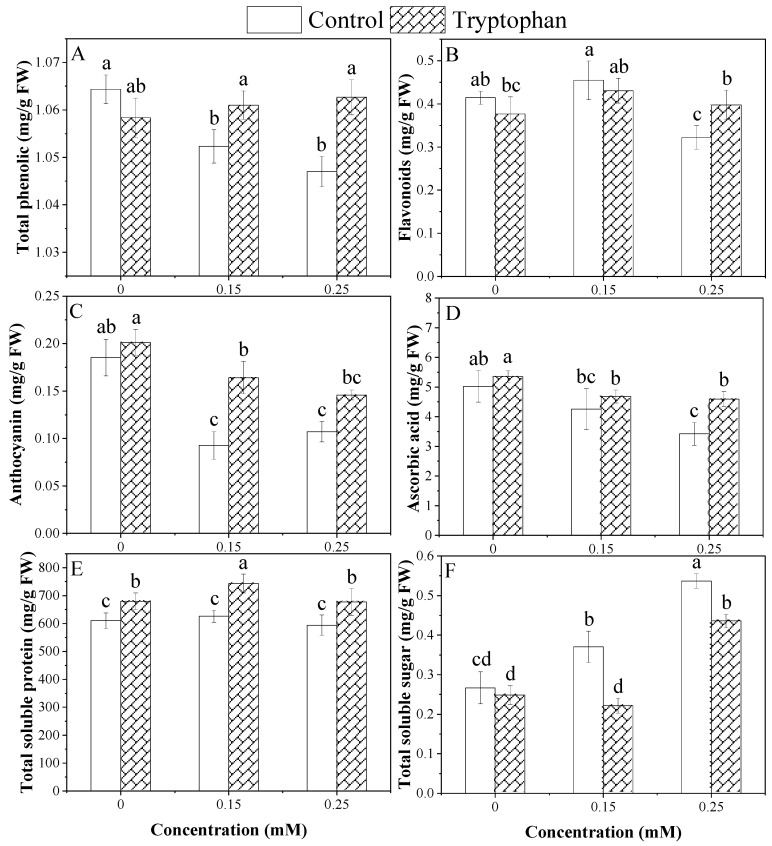
Comparative change in biochemical parameters under cadmium stress levels (0, 0.15, 0.25 mM) with tryptophan (1%) seed treatment (**A**–**F**). Error bars indicate standard deviation, and different letters indicate significant difference at *p* < 0.05 by LSD test.

The effect of Cd stress level on phenolic contents was non-significant, and a significant effect was observed by tryptophan seed treatment. The interaction between the two factors was also significant. There was a negligible effect of tryptophan seed treatment and stress upon this attribute. Tryptophan seed treatment significantly increased the phenolic contents under both Cd levels, and it was decreased without tryptophan seed treatments as compared to controlled conditions, but that difference was negligible and statistically at par (Figure 5A). Cadmium stress at the higher level (0.25 mM) decreased the flavonoid contents of plants, while it was highest at the lower level (0.15 mM) of Cd stress. The tryptophan seed treatment effect was found to be positive in alleviating stress in higher Cd levels. However, tryptophan seed treatment had a negligible effect on the lower stress and control levels. Data analysis showed the substantial effect of the level of stress and a non-significant effect for the tryptophan seed treatment. Moreover, a significant interaction between these two factors was observed (Figure 5B). Anthocyanin was significantly decreased by Cd levels (0.15 and 0.25 mM) compared to no stress level. In contrast, the difference between lower and higher Cd levels was insignificant under the tryptophan seed treatment. Tryptophan seed treatment significantly increased the anthocyanin in both Cd levels as compared to the control, and it was found to be higher in 0.15 mM Cd compared to 0.25 mM of Cd level, as shown in Figure 5C. Ascorbic acid was significantly affected by the Cd stress compared to no stress, but the difference between the two Cd levels was indifferent. Tryptophan seed treatment increased and maintained the ascorbic acid in the sunflower plant under both Cd stress levels (0.15, 0.25 mM). Soluble sugar contents were found to be higher under the higher Cd level (0.25), while it was lower under the Cd level of 0.15 mM when tryptophan seed treatment was applied. Tryptophan seed treatment decreased the soluble sugar contents in both Cd levels (Figure 5F). A highly significant difference was noted for tryptophan seed treatment effect and Cd stress, and the interaction between these two factors was also significant for the soluble sugar contents in sunflower plants (Table 3). As for as concerning total soluble proteins, Cd stress levels had a significant effect and tryptophan seed treatment had highly substantial effects. Moreover, a non-significant interaction between these two factors was observed. The untreated plants exhibited a decreased value of total soluble protein content under Cd stress conditions, and the sunflower was marked with the increased value of this attribute by tryptophan seed treatment. The highest increase in total soluble protein was noticed under a Cd stress level of 0.15 mM with tryptophan seed treatment, as shown in Figure 5E.

## 4. Discussion

Heavy metals are naturally present in the Earth’s crust and found in almost every environmental matrix. Soil heavy metal pollution has become a global environmental issue that has grabbed the public interest of people concerned with environmental issues [46]. Cadmium is a persistent non-essential and non-biodegradable element for plant excrescence [47]. Different methods enhance plant production under Cd-stressed and non-stressed circumstances [48,49]. Tryptophan seed treatment improves plant growth under both stress and non-stress conditions.

The rate of seed germination is an indicator of vigor. Seed germination is significantly affected by cadmium. However, the effects differed depending on the cultivar [50]. Catiempo et al. [51] determined that seedling establishment and the quality and yield of sunflowers was mainly influenced using a tryptophan seed treatment technique. In sunflower plants, data also showed a significant effect of Cd stress on seed germination in both conditions (control and tryptophan). The current study found similar results where tryptophan seed treatment improved seed germination under stress and non-stress conditions (Figure 3F). In a previous report, it was concluded that the growth of the roots, root biomass, length, surface area and volume, as well as the number of root tips and forks, was stimulated by Cd. Even while low doses of Cd significantly affected the growth of roots, decreased shoot biomass and higher MDA content in the shoots [52]. In our research, the data also revealed that Cd stress substantially impacts sunflower plant growth. Root and shoot length and biomass decreased with increasing Cd stress and MDA levels (Figure 4A). Tryptophan seed treatment could affect sunflower plant morphology and physiology under stress. Sunflower is generally considered a fast-growing crop and stress-tolerant to heavy metal stress [53,54]. In our experiment, the results showed that root and shoot length was negatively affected by Cd stress. However, the application of tryptophan increased shoot length in both stress conditions, as was previously observed by El-Sayed et al. [55]. Similarly, in our experiment, the negative effect of Cd stress was observed, and it was the minimum in lower (0.15 mM) and maximum in higher (0.25 mM) Cd stress levels. Tryptophan seed treatment increased the root and shoot length and biomass under both stress levels of Cd. Tryptophan seed treatment under Cd stress conditions also showed a substantial improvement in growth, number of leaves, and fresh and dry biomass of sunflower leaves (Figure 1). Strong evidence from recent studies has shown the potential role of tryptophan seed treatment in promoting the growth of sunflower seedlings under Cd stress. A boost in the improvement of leaf membrane stability, leaf area, and the number of leaves per plant was observed using a tryptophan seed treatment [56].

The plant pigment content, metabolic parameters, and heavy metal uptake (*Brassica jounces* L.) varied in cadmium stress. The plant’s growth, chlorophyll content, and carotenoids were negatively affected by Cd stress [57]. In this study, the growth parameters and biochemical components decreased in sunflower plants under Cd stress. However, cadmium stress (0.15 and 0.25 mM) significantly reduced the carotenoid contents of sunflower plants. As regards the effect of tryptophan, it was found positive at alleviating stress. The protein contents also decreased [57]. Chlorophyll *a*, chlorophyll *b*, chlorophyll *a*/*b* ratio, total chlorophyll, and total protein contents also decreased in the sunflower plants. However, tryptophan was found to reduce the Cd effect and increase the carotenoid contents (Figure 2F). Previous literature clarified that amino acids can form complexes with metal ions, notably Zn, through the carboxyl group to increase their bioavailability as treatment agents [58]. Zinc protects plants from many stressors by boosting antioxidant activity and detoxifying reactive oxygen species [59]. An essential zinc vitamin prevents Cd absorption [60]. Glutamate serves as a growth regulator and a signaling agent. Lysine is a crucial precursor to glutamate [61]. Lysine also plays a role in other essential processes in plants like lipid metabolism, proline biosynthesis and starch control [62]. Zn–Lysine seed treatment thus increases the plant’s yield, photosynthetic capacity and nutrient content. Lysine also showed many crucial plant metabolic processes, including lipid metabolism, glycolysis, tryptophan, nucleotide, TCA cycle, proline biosynthesis, and starch control [62]. Treatment of Zn–Lysine, therefore, increases plant production, photosynthetic capacity and nutritional levels [63]. Similarly, the current study reveals that tryptophan seed treatment suppressed the effect of Cd stress on the sunflower plant. It was noticed that the photosynthetic contents were improved under various levels of Cd stress. The sharp decline in chlorophyll pigments and increased carotenoid content was specific and may have been related to Cd tolerance capacity. Due to Cd stress, chlorophyll synthesis in both conditions was reduced with sunflowers showing the pigment decline. By restricting the intake of vital elements, Cd like other metals, substantially affects plant photosynthetic pigments and replaces the magnesium position in the chlorophyll structure [64]. However, the use of tryptophan improved photosynthesis in both stressed and non-stressed conditions by increasing the chlorophyll contents (Figure 2). This could be due to the tryptophan seed treatment increasing plant nutrient uptake and decreasing wheat root Cd absorption [65]. Aside from that, according to [66], amino acids encourage plants to produce more chlorophyll.

In the current study, morphological attributes exhibited significant reductions under Cd stress. The morphological and growth characteristic decreases were specific in both conditions and may have been related to Cd tolerance capacity. Under Cd stress, growth may be reduced due to Cd-induced oxidative damage, reduction in the photosynthetic process, and reduced nutrient contents. However, the tryptophan seed treatment improved these attributes in sunflower plants. A tryptophan seed treatment with similar positive results was also noted previously [56]. A tryptophan seed treatment could shield plants from the harmful effects of Cd stress and enhance the amount of individual phenolics under stress (Figure 4A). Flavonoids were increased in sunflower plants under the lower Cd stress (0.15 mM) and decreased at the high stress level. The effect of the seed treatment gave the benefit of plant growth under a higher level of Cd stress (Figure 5B). This result was found to be in agreement with Mahmood et al. [67], who concluded that, under Cd stress, plants experienced higher MDA levels, despite enhanced phenolic and flavonoid deposition; a number of growth and yield markers were still ineffective in mungbean plants.

In the present research, Cd mainly increased the peroxidation of lipids in the form of H_2_O_2_ levels. It was discovered that highly (0.25 mM) stressed plants suffer from more oxidative stress than low stress (0.15 mM) due to increased levels of H_2_O_2_ content, which could be a result of greater Cd retention in sunflowers, as evidenced by the data (Figure 4). Our results concurred with those of [68], who found that plants exposed to Cd toxicity suffer from lipid peroxidation, which negatively affects plant growth. Plants raised from the seed-primed application have a lower MDA and H_2_O_2_ under both Cd stress levels (Figure 4A,B). Previous research found that Zn–Lysine decreased MDA and H_2_O_2_ levels in radish spinach [69] and maize under heavy metal and abiotic stress [63]. Under regulated and stressful conditions, Zn–Lysine-activated antioxidant defense mechanisms may be linked to decreased levels of oxidative stress. Under Cd toxicity, Zn–Lysine showed promise in wheat cultivars and lowered Cd uptake [47]. The outcomes of the current study reveal that primed sunflower plants had a more robust antioxidative defense system associated with lesser uptake of Cd to other plant parts when compared to the stress and non-primed plants, improving plant tolerance to Cd. The present study enhanced the antioxidant activities in both conditions as Cd levels increased (Figure 4 and Figure 5B). Similar increases in antioxidant activity were observed in different plant species, including wheat [70]. The results of our study explained that the plant grown with tryptophan seed treatment had much lower MDA levels than untreated stressed plants. Similar conclusions were recorded by [69], where amino acid lessened the harmful impacts of Cd on cereal crops by reducing antioxidant activity.

In the present studies, the most significant decrease in plant biochemical contents was observed in both stress conditions under Cd but reduced its effect by tryptophan seed treatment. The lower biochemical contents in sunflowers may be due to higher Cd uptake. Byeon and Back [71] discovered that cadmium stress decreased nutritional content. However, plants grown from the administration of tryptophan exhibited higher levels of nutritional content in both stressed and unstressed situations. Previous studies have demonstrated that tryptophan increased K, Na, and Ca levels in diverse crops, such as wheat under metal stress [71]. Thus, we understand that Cd toxicity decreased sunflower growth by affecting physio-morphological parameters. The tryptophan seed treatment enhanced plant growth by increasing germination, photosynthetic activity, and biochemical attributes. However, to better understand the effects of exogenous tryptophan on sunflower plant physiology and production, more comprehensive studies should be performed, not only on growing seedlings from tryptophan-treated seeds but also on the establishment of plants in field systems.

## 5. Conclusions

In this study, we evaluated the effects of a tryptophan seed treatment under varying levels of Cd toxicity. Cd stress affects a plant’s morphological traits, photosynthetic pigments, reactive oxygen species, and biochemical attributes. However, using tryptophan as a seed treatment agent significantly alleviated Cd toxicity and enhanced plant metabolites and photosynthetic capacity, with preferential limitations in the accumulation of MDA and H_2_O_2_ in sunflower plants. Cd stress levels (0.15, 0.25 mM) decreased the morphological attributes (shoot and root length, dry and fresh weight, average leaf area, and seed germination). Higher levels of Cd stress (0.25 mM) decreased photosynthetic pigments and plant metabolites while enhancing the MDA, H_2_O_2_, and soluble sugar contents. The tryptophan seed treatment lessened the effect of Cd on plant growth and promoted root and shoot length, root and shoot fresh weight, seed germination, increased the average leaf area, and enhanced the carotenoid contents, total phenolics, flavonoids, ascorbic acid, soluble proteins, and anthocyanins compared to controlled conditions under Cd levels (0.15 mM and 0.25 mM). In conclusion, these findings suggest that tryptophan can significantly reduce the adverse effects of Cd on sunflower growth, which can be used as a seed treatment to mitigate Cd pollution. The current study also paves the research pathway for further investigation into the impact of tryptophan on the expression of stress-related genes in arable crops.

## Figures and Tables

**Table 1:** ANOVA for the summary of growth characteristics and photosynthetic pigments of sunflowers grown under the cadmium levels (0, 0.15 mM, 0.25 mM) with tryptophan seed treatment.

Treatments	Root Length (cm)	Shoot Length (cm)	Root Fresh Weight (g)	Shoot Fresh Weight (g)	Root Dry Weight (g)	Shoot Dry Weight (g)	Chl *a* (mg/g FW)	Chl *b* (mg/g FW)	Chl *a*/*b* (mg/g FW)	Total Chl (mg/g FW)	Total Carotenoid (mg/g FW)
ANOVA											
Cd Stress (A)	***	***	***	***	***	***	NS	***	***	***	***
Tryptophan (B)	NS	*	***	*	***	*	***	**	NS	*	***
A × B	NS	**	NS	NS	NS	NS	**	***	***	***	**

NS is non-significant; *, **, and *** show the level of significant results at *p* ≤ 0.05, 0.01, and 0.001, respectively, followed by the LSD test.

**Table 2:** Analysis of variance for the summary of germination and leaf parameters of sunflowers grown under the cadmium levels (0, 0.15 mM, 0.25 mM) with tryptophan seed treatment.

Treatments	Seed Germination	No. of Leaves/Plant	Average Leaf Area (cm^2^)	Average Leaf Length (cm)	Average Leaf Fresh Weight (g)	Average Leaf Dry Weight (g)
ANOVA						
Cd Stress (A)	**	***	**	*	*	*
Tryptophan (B)	*	*	*	*	*	*
A × B	NS	**	NS	NS	NS	NS

NS is non-significant; *, **, and *** show the level of significant results at *p* ≤ 0.05, 0.01, and 0.001, respectively, followed by the LSD test.

**Table 3:** Analysis of variance for the summary of biochemical attributes of sunflowers grown under the cadmium levels (0, 0.15 mM, 0.25 mM) with tryptophan seed treatment.

Treatments	MDA (µmol/mL FW)	H_2_O_2_ (µmol/mL FW)	Total Phenolic (mg/g FW)	Total Flavonoid (mg/g FW)	Anthocyanin (mg/g FW)	Ascorbic Acid (mg/g FW)	Total Soluble Protein (mg/g FW)	Total Soluble Sugar (mg/g FW)
ANOVA								
Cd Stress (A)	***	NS	NS	**	***	**	**	***
Tryptophan (B)	***	NS	**	NS	***	**	***	***
A × B	**	*	*	*	*	*	NS	*

NS is non-significant; *, **, and *** show the level of significant results at *p* ≤ 0.05, 0.01, and 0.001, respectively, followed by LSD test.

## Data Availability

The authors declare that data supporting the findings of this study are available on request from the corresponding author.
